# Unified framework for the ingestion of early epidemic data for downstream data analytics

**DOI:** 10.12688/wellcomeopenres.24776.1

**Published:** 2025-09-22

**Authors:** Everlyn Kamau, Sadie Kelly, Dhruv Darji, Amrish Y. Baidjoe, John S. Brownstein, Finlay Campbell, Abhishek Dasgupta, Marie-Amélie Degail, Anastasiia Demidova, Luca Ferretti, Aimee Han, Stephen Leshan Koyie, Patricia Ndumbi Ngamala, Olivier Le Polain, Amanda Rojek, Jacquelin Sauer, Samuel V. Scarpino, Kara Sewalk, Juliana Sopko, Stanislaw Zakrzewski, Laura Merson, Moritz U. G. Kraemer

**Affiliations:** 1 F.I. Proctor Foundation, University of California San Francisco, San Francisco, California, USA; 2 Department of Biology, University of Oxford, Oxford, England, UK; 3 Nuffield Department of Medicine, University of Oxford, Oxford, England, UK; 4 Pandemic Sciences Institute, University of Oxford, Oxford, England, UK; 5 Operational Centre Brussels (OCB), Medecins Sans Frontieres, Brussels, Belgium; 6 Luxembourg Operational Research Unit (LuxOR), Médecins Sans Frontières, Luxembourg, Luxembourg; 7 Computational Epidemiology Lab, Boston Children’s Hospital, Boston, USA; 8 Harvard Medical School, Boston, Massachusetts, USA; 9 WHO Hub for Pandemic and Epidemic Intelligence, Berlin, Germany; 10 Research Software Engineering Group, University of Oxford, Oxford, England, UK; 11 WHO, Geneva, Switzerland; 12 Independent Researcher, London, UK; 13 Department of Community Health Sciences, Boston University School of Public Health, Boston, Massachusetts, USA; 14 Institute for Experiential AI, Northeastern University, Boston, Massachusetts, USA; 15 Department of Public Health and Health Sciences, Northeastern University, Boston, USA; 16 Santa Fe Institute, Santa Fe, New Mexico, USA; 17 Technical University of Lodz, Lodz, Poland; 18 Institut Pasteur de Dakar, Dakar, Dakar Region, Senegal

**Keywords:** Epidemic, Outbreak, Data Schema, Toolkits, Reporting Guidelines

## Abstract

**Background:**

Early-phase data during an epidemic are often heterogeneous and difficult to integrate across systems, therefore a need for standard tools and reporting guidelines to facilitate timely and reliable data collection. The Global.health team developed a core data schema for the ingestion of epidemic data, allowing interoperability where data curated to this schema are readily ingested into existing systems for analysis.

**Methods:**

We review the Global.health data schema against the WHO T0 and T1 outbreak toolkits for consistency. We conducted literature review to identify epidemiological parameters and questions for clinical and public health response during early epidemic investigations and linked these with associated data items to determine the minimum requirements for reporting. We also examined existing epidemic reports to evaluate data requirement for parameter estimation versus data availability.

**Results:**

We identified eight digital tools and data toolkits used for epidemic evaluation and field investigation, but most are tailored to specific diseases. We extracted 78 key epidemiological parameters from the literature review and organized them into eleven categories considered important for epidemic investigation. We highlight the minimum data reporting requirements for estimation of key epidemiological parameters, for example, the date of symptom onset would be required for estimating incubation period and reproduction number, while household size would be required for estimating serial interval distribution. We determined that about 30% of the data variables in the Global.health data schema are likely to be prioritized for data collection, while some information related to vaccination; travel history and medical treatment were less likely to be available.

**Conclusions:**

The low availability of some data variables is likely to impact public health response and improved data collection should prioritize those variables. The output here will be useful to develop data collection and reporting guidelines during the early phase (first 100 days) of an epidemic.

## Introduction

The first weeks of an epidemic are crucial for mounting an effective response. During this period, it is essential to obtain accurate estimates of key epidemiological parameters such as transmission rates and disease severity, both for known and newly emerging pathogens. This is also a critical time for official reporting systems to assess availability of resources, evaluate clinical impact, plan rapid testing and interventions (including assessing their effectiveness), monitor disease progression and estimate potential burden in the society. However, in the early stages of an epidemic, data on infectious disease cases and associated metadata – such as case and death counts, locations, sources of infection, laboratory tests performed, and vaccination status - are often sparse and inconsistently captured, thus hindering data integration, analysis and interpretation.

These limitations hinder the value of these data in public health decision making, despite the fact they may otherwise be critically informative ^1^. Heterogeneity can result from variation in content, quality, volume, format, veracity, completeness and standardization processes ^2^. Additionally, a lack of standardisation in data capture systems delays data integration for research and statistical analysis, which impedes understanding and forecasting of the trajectory of an epidemic ^3^. This limits real-time use of data to effectively assess and optimize public health operations during epidemic response ^4^.

Another key issue in understanding disease dynamics is data availability, which is limited by privacy concerns, regulatory restrictions, conflicts of interest, complex processes to arrange data sharing agreements between entities, and the absence of trust, particularly in the digital era ^1, 5^. Socio-technical factors such as language barriers, different regional and national structures and rules that govern dissemination of health information, and imbalances in technical capacities and power also impact the establishment of effective and meaningful data sharing ^6^.

The Global.health platform assembles and curates open-access infectious disease data to enable situational awareness and risk assessments on emerging epidemics for decision-makers, researchers and the public ^7^. Global.health recognizes a critical gap in the continuum of data during epidemics, particularly in the early phase when the opportunity for containment is highest. Global.health aims to make data sharing more efficient and data more openly accessible to communities and groups working on epidemic response. A main step towards this aim is data interoperability through a standardized data harmonization schema to ensure epidemiological data are collected in a uniform format that is easily shared for rapid analysis and determination of key epidemiological parameters. A crucial component of the schema development is defining the most important questions and parameters to be estimated during the early phase of an epidemic.

Here, we describe how we determined such questions and parameters through background research, literature review and expert interviews. We also describe the similarity and alignment of the Global.health data schema ^8^ with existing toolkits (T0 and T1 forms) designed by the World Health Organization (WHO) and highlight the minimum set of data items or variables required for epidemic investigation. Ultimately, we aim to create a standardized schema where the output of data curated to this schema can be shared in a format readily ingested by existing and future analytical systems for epidemic analytics.

## Methods

**Figure 1.**
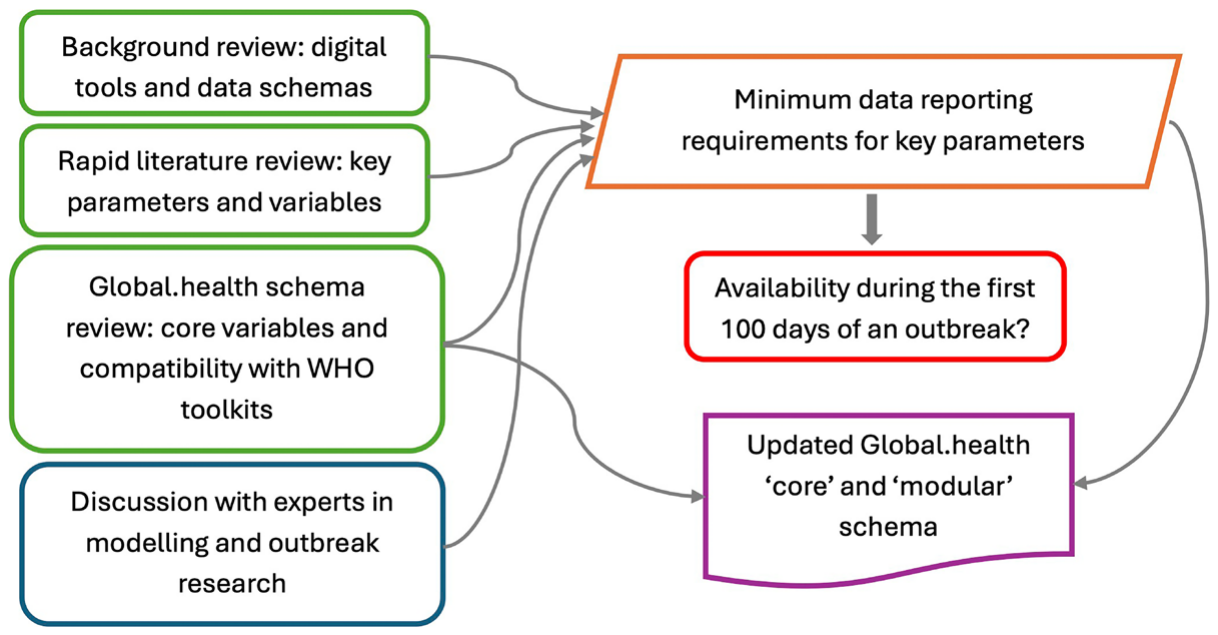
Conceptual framework of this study. Schematic illustrating the analyses and steps involved.

Our workflow and qualitative analysis are illustrated in Figure 1 and were executed as follows:

(i) *Background review of digital tools and data schemas for epidemic evaluation*. We first reviewed existing toolkits and data schemas for epidemic data collection guidelines through internet search and discussion with experts in various jurisdictions. Twenty-two scientists and academic researchers engaged in disease modelling in the US, UK and Canada; epidemiologists in national and international public health agencies (WHO offices in Geneva and Berlin and WHO regional office in Africa) and experts at Médecins Sans Frontières provided input. The discussion objectives were based on expert knowledge about resources for data and information collection with an emphasis on epidemic investigation.

(ii) *Rapid literature review of key parameters to be addressed during the first 100 days of an epidemic.* Next, we conducted a literature review to identify key epidemiological parameters or questions that must be addressed during the first 100 days of an infectious disease epidemic. We searched the PubMed database was searched using the following terms: (((((epidemiology) OR (epidemiological)) AND ((((((outbreak) OR (outbreaks)) OR (disease outbreak)) OR (epidemic)) OR (epidemics)) OR (pandemic*))) AND ((((parameter*) OR (question*)) OR (data)) OR (information))) AND (((early) OR (initial)) OR (priority))) AND ((((((((((incubation period) OR (case fatality rate)) OR (case fatality rate (CFR))) OR (risk factors)) OR (basic reproduction number)) OR (R _0_)) OR (effective reproduction number)) OR (serial interval)) OR (delay distributions)) OR (generation time)). The search was conducted on 23 ^rd^ June 2024 and restricted to articles available in English. The final set of articles was reviewed for eligibility by two independent reviewers. Other relevant grey literature which had been published on public health response of an infectious disease epidemic was identified through contacting the 22 experts mentioned above. We also reviewed the references cited in these relevant publications and grey literature to identify other eligible articles for full text review and data extraction.

(iii) *Review of Global.health data schema core variables.* We examined the variables that underlie the questions and responses in the Global.health day 0 data schema (which can be found at https://github.com/globaldothealth/outbreak-schema) against variables in existing toolkits (WHO’s Epi Core, T0 and T1) to ensure both systems are aligned, and data are captured similarly.

(iv) *Review of minimum data reporting requirements for key parameters.* The parameters and questions underlying the categories listed in (ii) and (iii) above were reviewed to identify the associated variables. We also reviewed the WHO outbreak toolkits and asked a core group of 10 individuals (of the 22 mentioned above) with expertise in modelling and epidemic research to suggest other suitable variables.

(v) *Assessment of availability of variables during the first 100 days of an epidemic.* We assessed data availability by conducting an assessment and rating of the likelihood of data variables being present in the Global.health data sources ^7^ during the first 100 days of an epidemic. Availability was determined as the count or frequency of records containing a variable of interest. We reviewed the current list of variables in the Global.health data schema which has been used for previous epidemics including Ebola (2018–2020), Marburg (2024), COVID-19 (2019–2023) and Mpox (2023–2024), and assigned to each variable a likelihood rating of ‘High’, ‘Medium’, or ‘Low’ based upon the team’s previous curation experience in epidemic data tracking. The ratings ‘High’, ‘Medium’, or ‘Low’, indicated availability of a variable in epidemic data counted as ≥80%, ≥50% to <80% and <50%, respectively. The likelihood of availability was considered for the first 100 days of an epidemic, when possibly more detailed individual-level cases may be reported, as opposed to a later time in an epidemic where reports tend towards aggregate summaries. We note that the current Global.health schema was amended or updated from the initial schema used in the epidemics listed above, following alignment with the WHO toolkits.

(vi) *Updating the core schema*. Based on the output from (iii) and (v) above, we updated the Global.health ‘core’ schema to ensure it captured variables relevant to all epidemic types and added a ‘modular’ schema to contain variables related to exposure, treatments and vaccination which can be adjusted to become pathogen-specific. The modules in the modular schema are pre-designed and can be activated rapidly once an epidemic is declared. The exposure variables were adapted from WHO’s T0/T1 and disease-specific toolkits to capture additional detail where available.

## Results

### Background review of digital tools and data schemas for epidemic evaluation

We identified several toolkits and data schemas as listed in Table 1. Six out of eight of these resources were designed to ingest data from epidemic field investigations.

**Table 1.** Toolkits. Summary of existing toolkits and digital resources for infectious disease data and information collection. (WHO: World Health Organization; CDC: Centers for Disease Control).

Digital tool	Brief description of purpose, source and design
WHO T0 case investigation form ^10^	The initial generic form designed to help outbreak field investigators rapidly understand an epidemic and propose initial control measures. It supports the collection of the minimum Epi Core ^11^ variables for epidemic investigation ^12^, and was designed to help describe the epidemic over time, geographical spread and persons affected to guide decisions regarding the first measures to control the epidemic. Data collected from the WHO T0 case investigation form ^10^ can also be used to generate hypotheses about the case, source, and transmission mode of a pathogen. The additional variables collected on the T0 form outside of the specified Epi Core variables were collated from the most common variables indicated on the WHO Disease outbreak toolkits ^13^, which were created to enable rapid specification of a case definition, collection of appropriate data for the disease and for field workers to have a resource for tools and training.
WHO T1 case investigation form for outbreaks of unknown cause ^10^	Used to collect detailed information on any situation involving an epidemic of unknown origin. It is designed for initial data collection to identify the epidemic source, mode of transmission or agent involved, and is composed of 462 variables organized in five categories, many of which are conditional or symptom-based checklists. The clinical variables in the WHO T1 form are grouped per functional body system (e.g., neurological, digestive, cutaneous), which is preferred as opposed to the syndromic approach when the disease is unidentified ^10^. The exposure variables in the WHO T1 form support the investigation of risks related to transmission, food or water contamination with pathogens and environmental toxins and hazards (e.g., heavy metals) that can lead to an outbreak.
The European Surveillance System (TESSy)	The TESSy portal facilitates collection, analysis, and dissemination of indicator- and event-based surveillance data in infectious disease and associated health issues. The TESSy metadata set lists the collected data for a wide range of pathogens, from both aggregate and case-based data. The_European Surveillance System (TESSy), is now integrated into EpiPulse with the five_Epidemic Intelligence Information System (EPIS) platforms and the_Threat Tracking Tool (TTT) ^14– 17^. TESSy’s standardised data formats and validation rules are provided within the metadata to improve data quality, but the rationale for the collection of each variable is not provided. Data sources include those that are open access such as FluTrackers, monitoring websites and trusted media sites, as well as WHO sources and other access-restricted sources. Furthermore, access to EpiPulse is restricted.
The article ‘Key data for outbreak evaluation: building on the Ebola experience’ ^18^	This draws from the authors prior experiences in outbreak data collection and analysis and provides a checklist of data needed to quantify severity and transmissibility, characterize heterogeneities in transmission and their determinants, and assess the effectiveness of different interventions. The article recommends that data are differentiated into individual-level data, exposure data, and population-level data, and the checklist also highlights the potential issues and biases that can be present in these data. The setting for data capture in the article is also primarily field investigations, like the WHO toolkits.
WHO PISA (Pandemic Influenza Severity Assessment)	PISA details the requirements for influenza surveillance and how to assess the impact of a potential outbreak ^19^. It focuses on assessment of (i) transmissibility using case incidence and positive test rates, (ii) severity using deaths and hospitalizations, and (iii) impact using incidence, excess pneumonia/influenza or all-cause mortality, confirmed cases, hospital admissions, absenteeism in school and workplaces, and healthcare resources use such as beds occupied.
CDC framework for assessing epidemiological effects of influenza epidemics and pandemics ^20^	Similar to the WHO’s PISA risk assessment on transmissibility and severity. Transmissibility is assessed using R _0_, the serial interval and the attack rate, while severity is assessed from case fatality, hospitalization records and genetic markers of virulence. This document also lists the strengths and limitations of each parameter and provides considerations towards evaluation of data quality. The WHO PISA suggests that parameters should be reviewed by age groups, and severity should consider presence or absence of underlying chronic diseases.
Key indicators for WHO priority pathogens with a confirmed outbreak	WHO’s key epidemiological indicators and recommended analyses for surveillance of priority pathogens with a confirmed outbreak have been published for various diseases including cholera ^21^, measles, yellow fever and meningitis ^22, 23^. They focus on incidence, attack rate, severity (case fatality) and test positivity. Only for cholera have the indicators been linked to specific data points and rationale provided.
Global.health day 0 core schema	The Global.health day 0 core schema ^8^ uses a common data model to standardize key epidemiological epidemic data that includes details for case status, location, age, sex, symptoms, transmission, hospitalization, treatment, vaccination, outcome, contacts, travel history, occupation, genomics and more. Each individual case is assigned a unique identification number when added to a line list and data are de-identified to protect individual privacy. The Global.health data sources are primarily those that are publicly available. Originally designed in the context of COVID-19 data curation ^24^ and most recently updated for mpox reporting (covering the 2022 global epidemic) where it was based heavily on the WHO mpox case report form. It collects data and metadata from official and non-official online public sources and provides the original source(s) of information for each case to support transparency and data sharing. These variables are specified by minimum case requirements and are pathogen-agnostic.

### Literature review of key parameters to be addressed during the first 100 days of an epidemic

**Figure 2.**
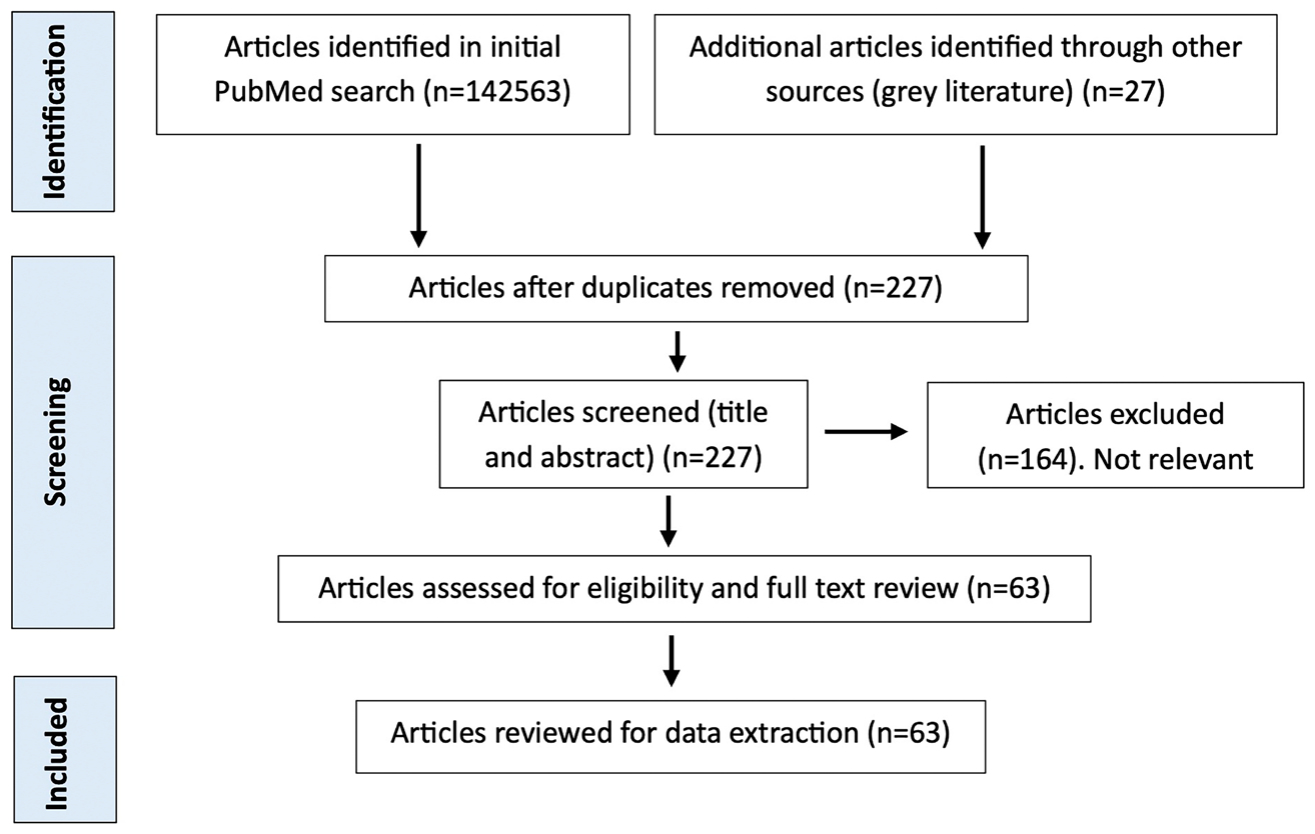
Literature search. Literature search workflow to identify and extract key questions and parameters analyzed to guide public health response during the early stages of an epidemic or outbreak.

Our search terms identified 142,563 potentially relevant articles. We removed duplicates resulting in 197 articles for title and abstract screening ( Figure 2). Of the 197, 164 were irrelevant for the purpose of the study and excluded and the remaining 35 were evaluated for full-text eligibility. Thirty-five other publications and grey literature addressing crucial epidemiological questions early in an epidemic, but not captured in our initial search, were also reviewed. In total, 62 articles were reviewed for data extraction. We identified and extracted 78 key epidemiological questions or parameters from the literature review and organized them into eleven categories as described in Table 2. The questions and categories were shared with a panel of experts in infectious disease outbreak epidemiology for suggestions and comments on suitability for addressing questions during a public health response. The categories were based on prior work by Perrocheau *et al.* ^9^ and with an aim of developing a consensus on the most important parameters or questions that should be answered during the early stages of an infectious disease epidemic.

**Table 2.** Key categories and parameters. A summary of categories and parameters or variables considered important for infectious disease epidemic investigation.

Category	Description and some of the parameters included
Epidemiological parameters	Used to model transmission and predict epidemic growth rate. Include basic reproduction number, incidence rate, effective reproduction number, incubation period, serial interval, generation time and secondary attack rate.
Delay distributions	Describe delays between events in the infection history and reporting, which affect transmission and can have implications for epidemic control.
Disease severity factors	Include case fatality rate, hospital and intensive care unit admission rates.
Risk factors for epidemic growth, susceptibility to infection and poor outcomes	Include individual level demographic factors, medical factors (e.g., pre-existing conditions, vaccination history, reinfections), occupational factors (e.g., health care worker status or other risks from specified occupations depending on the pathogen), behavioral factors (e.g., details of exposure and travel history to determine settings and risk factors of transmission).
Where/Who factors	Important for inferring transmission heterogeneities or risk of infection across different locations or population groups.
Clinical features	Case defining or prognostic features, laboratory results and vital signs.
Contextual and external factors	Broader population-level indicators for surveillance bias (how an infected case was identified to the healthcare system), impact of human mobility patterns, climate and other drivers of transmission, as well as knowledge of pathogen genomic lineage for detailed analyses.
Diagnostic factors	Knowledge of testing methods and strategies across different locations to interpret incidence and other epidemiological aspects listed above.
Surveillance parameters	High-level estimates of the proportion of infections reported, asymptomatic cases as well as peak-time forecasts of incidence, useful to model the impact of the epidemic. These could be a function of, or derived from, the contextual and external factors.
Pharmaceutical interventions	Medical treatments and vaccinations, and their impact on incidence and individual outcomes.
Non-pharmaceutical interventions	Non-pharmaceutical interventions, or “Public Health and Social Measures” ^25^, such as isolation procedures, and other behavioral interventions (e.g. hand-washing, mask-wearing) and their impact on incidence and individual outcomes.

### Global.health data schema core variables

We reviewed the Global.health day 0 data schema ^8^ and categorized the associated core variables into six main categories based on the structures in the WHO’s Epi Core, T0 and T1 toolkits. This ensures variables in both systems are aligned and data are captured similarly. The categories are as follows:

I. *Demographics*: includes variables with responses such as age, gender, sex at birth, case status (e.g., confirmed, suspected, probable), locality or residence location and job occupation. The residence query results in a location structure that is pre-filled by mapbox with latitude and longitude, ISO code for country and geographical administrative information.
II. *Medical history*: this category contains variables on pre-existing conditions including pregnancy, co-infections, history of the same infection prior to the current diagnosis and vaccination information.
III. *Clinical presentation*: includes variables on reported symptoms, date of symptom onset, hospitalization, intensive care treatment, outcome of illness (e.g., death, recovered, post-acute sequelae), vital measurement results, pharmaceutical and non-pharmaceutical treatments.
IV. *Laboratory Information*: including genomic information, testing information (e.g., type of specimen, method of testing date of sample collection, pathogen strain/subtype) and date of case confirmation.
V. *Exposure*: contains variables indicating likely sources of exposure e.g., contact with suspected or confirmed cases or infected animals/animal products, or through environmental exposure (water sources, chemicals, exposure).
VI. *Source Information*: contains variables on data origin (e.g., government bulletins, ministry of health reports, media platforms, etc.), date of entry and/or modification and curator’s details and comments.

Several variable responses in the Global.health schema e.g., symptoms, specimen type, pre-existing medical conditions and type of medical treatment can be selected from predefined lists with an option for free text entry.

### Minimum data reporting requirements for key parameters

We identified data variables associated with the key parameters and questions ( Table 3) and considered these as the minimum data requirements. Additionally, we reviewed the data reporting requirements of the International Health Regulations (IHR, 2005) and the variables in the Global.health schema that relate to these requirements were flagged to ensure that data collected in the schema is appropriate for the IHR reporting requirements.

**Table 3.** Minimum data requirements. This table highlights variables or information that would be considered the minimum requirements for various categories and parameters.

Category	Parameter(s)	Minimum variables or information required
Epidemiological parameters	Incubation period ^a^	Date of symptom onset. Exposure date or date of last contact to the suspected source (e.g., animal, infectious individual, toxin, etc.) Travel dates (e.g., exposure interval at the source and symptom onset in other location).
	Basic reproduction number (R0) ^b^ Effective reproduction number (Rt) ^c^	Date of symptom onset of confirmed cases. Date of confirmation. Additional information on vaccination is recommended for *Rt* where suitable.
	Incidence rate ^d^, attack rate ^e^, growth rate ^f^	Knowledge of case status (e.g., ‘confirmed, probable, suspected’), location and date of case report.
	Secondary attack rate	Date of symptom onset in *infector* and *infected*
	Serial interval distribution ^g^	Household size Other supplementary data elements include travel history location(s) and setting where contact occurred with the confirmed/suspected case
Delay distributions	Measures of delays between events in the infection history and reporting (e.g., Date of symptom onset to date of reporting)	Date of symptom onset, date of case report, date of isolation, date of hospitalization, date of hospital discharge, outcome (e.g., death, recovery), date of outcome evaluation, date of specimen collection and date of case confirmation
Diagnostic factors	Diagnostic performance	Diagnostic test Results from negative and positive control samples Type of specimen
Impact of non-pharmaceutical interventions	Impact on incidence and individual outcome	Type of interventions and date Date of symptom onset, date of isolation, outcomes (hospitalization, death, recovery) Time series of suspected, probable or confirmed cases (population level)
Impact of pharmaceutical interventions	Impact on incidence and individual outcome	Medical treatments and vaccination (including timing) Outcomes (death, recovery, length of hospital stay)
Risk factors for epidemic growth, susceptibility to infection and individual outcomes.	Demographic, medical and occupation risk factors	Travel history location(s), date of country entry, and date when began travel, date of symptom onset, age, sex at birth, date of hospitalization, pre-existing conditions including pregnancy, presenting symptoms, treatments, vaccination. Occupation (e.g., healthcare worker), contact with infected or suspected case? close proximity with animals or insect bites prior to symptom onset? exposure to harmful water or chemicals? visiting a healthcare facility days prior to symptom onset? Infection history (previous similar infection)
Disease severity factors	Measure of severity e.g., Infection fatality ratio/Infection fatality rate, case-hospitalization ratio	Case status (e.g., confirmed, probable, suspected), disease outcome and date of outcome evaluation (hospitalization, death, recovery) Age, race, ethnicity, clinical vulnerability, was the case a healthcare worker? date of case report, intensive care treatment?

^a^ The interval between exposure and initial occurrence of signs and symptoms. ^b^ The expected number of cases generated by a single case in a population where all individuals are susceptible to infection. ^c^ The number of cases generated in the current state of a population. ^d^ The number of new cases in a population within a specified period of time. ^e^ The proportion of an at-risk population that contracts the disease during a specified time interval. ^f^ How quickly the numbers of infections are changing over a period of time. ^g^ The interval between symptom onset in an index case and in a secondary case.

### Availability of variables during the first 100 days of an epidemic

Of the current 140 variables in the Global.health data schema, 42 had a high likelihood of being captured in a line list in early epidemic data (Supplementary Table S1). These variables included parameters that describe pathogen name, case status, location of the case report, list of symptoms, date of case report, outcome, date of case confirmation, date of outcome evaluation, occupation, where contact with infected or suspected cases occurred, travel history and sources of information. Where travel history is mentioned, the travel location is indicated.

There were 29 variables with a medium likelihood of availability in epidemic data sources including pre-existing conditions, vaccination information, date of symptom onset, pathogen strain subtype, diagnostic test, contact with animal or insect bites, date of entry into the country, type of medical treatment and exposure to water or chemical agents (Supplementary Table S1). Other variables (n=56) were rated as low likelihood of availability. These included confirmed prior infection, co-infection with other pathogens, number of vaccine doses, date of first vaccination, admission to intensive care unit, date of isolation, vital measurement results, dates of medical treatment, type of specimen and mode of travel.

The current Global.health data reports are also less likely to answer whether or not an individual visited a healthcare facility several days prior to symptom onset, or how a case was found (which might help to assess surveillance bias), as well as reliably tell the date of the healthcare visit. The reports cannot reliably differentiate between primary and secondary cases within contact exposure, but where death and treatment are mentioned, the date of death and type of treatment are often indicated. Other things to note: (i) vaccination information is available depending on the causative pathogen and hence the low likelihood of availability as noted above, (ii) ‘death’ is the most commonly reported outcome while recovery or date of recovery is not always mentioned, (iii) where symptoms may not be mentioned, an asymptomatic case might be assumed, (iv) home monitoring may not be distinguished from the general ‘isolation’ variable, and (v) pre-existing conditions are reported more frequently than previous infections. The current Global.health schema variables on mass gatherings were specific to COVID-19 and therefore unlikely to be available for other epidemics.

### Open access and machine-readable data schema

Based on the results above, we updated the Global.health ‘core’ schema to capture variables relevant to all epidemic types and added a ‘modular’ schema. The latter contains two modules with variables related to exposure and interventions e.g., treatments and vaccination, which can be adjusted to become pathogen specific ( Table 4). The variables in both schema setups were reviewed against existing toolkits in terms of data type, question text, and response options to ensure consistency and interoperability with other data capture specifications. The schemas specify syntactic and semantic standard codes for each variable according to the WHO SMART guidelines ^26^, to allow for interoperability of shared data. These codes were prepared from standardised dictionaries including SNOMED-CT (International) ^9^, SNOMED-GPS (Global Patient Set) ^27^, LOINC ^28^, ICD-11 ^29^, ICHI ^30^ and ICF ^31^, and align with existing codes used in other WHO digital adaptation kits for interoperability within WHO systems. For each variable, multiple codes were specified where applicable to allow better interoperability among users or systems working with the resulting curated dataset. Collectively, the core and modular schema form a digital adaptation kit, which as part of the WHO SMART guidelines can be adapted to contain additional variables capturing exposure types, treatments, and vaccinations.

**Table 4.** Exposure and intervention modules. Each column represents the two Global.health schema modules. In each column we show the representative data elements and examples of specific variables related to these elements. Currently, the intervention module has only three data elements.

Exposure module	Intervention module
Contact with case (e.g., contact ID, contact setting, date of last contact)	Pharmaceutical interventions – vaccinations (e.g., number of doses, vaccine name, vaccination date, side effects)
Mass gathering (e.g., mass type, date of event, location of event)	Pharmaceutical interventions – treatments (e.g., type and name of treatment, route, start and end date, daily dose, traditional treatment)
Animal contact (e.g., animal species, date and location of contact with animal, insect bites or stings, contact with skinned wild game, raw animal meat of blood)	Non-pharmaceutical interventions (e.g., face mask, social distancing, hand washing, school closure)
Travel history (e.g., date of entry into country, location of travel, mode of travel)	
Treatment (e.g., visit to healthcare facility, type and location of facility, visit to a traditional healer)	
Water source (e.g., type of drinking water source, contact with flood water)	
Chemical source (e.g., potential source of chemical exposure, place and duration of exposure, suspected chemical product)	

## Discussion

Infectious disease surveillance requires timely and reliable data ingestion processes which are often complex and fragmented across institutions and governmental entities. Analytical methods, tools and resources for epidemic investigation have grown significantly over the last two decades, however, data acquisition and interoperability have remained a challenge and are difficult to standardize across pathogens and diseases. We have developed a unified data ingestion framework for descriptive mapping of variables collected during an epidemic response, which has a wide scope of parameters and variables preset with a uniform coding system for optimal interoperability. We examined the data collection variables in the Global.health data schema to assess interoperability with WHO systems and ensure that the output of data curated to the Global.health schema can be shared in a standard format readily ingested to support key epidemiological tasks identified by the WHO.

Currently, the occupational risk factors in the Global.health schema will differ depending on the type of epidemic, but it would be desirable to have agnostic occupation categories applicable to all epidemics. In our assessment of data availability for the minimum reporting requirements, we did not evaluate the likelihood of availability by geographical origin. This would be useful to identify gaps in data collection methods or surveillance system structures that could potentially challenge harmonizing surveillance data across countries.

The low availability of some data variables (Supplementary Table S1) is likely to impact epidemic and outbreak response and epidemiological parameter estimation. For instance, inadequate information on vaccination might hinder evaluation of coverage and impact immunization services on disease burden. Similarly, inadequate information on hospital discharge could hinder predictive models for estimating the discharge probability of acute care patients. We found that the date of specimen collection was lacking, which would also impact understanding of when a disease process was present in a patient. The ongoing efforts for improved data collection should emphasize such variables, and the several others in Supplementary Table S1, as they play an important role in monitoring disease response and management.

In future work, we will further assess whether the key parameters identified here can be adapted to the field resources available during an epidemic investigation through a user survey, the outcome of which will be used to update the core Global.health schema and the minimum data requirements for epidemic evaluation. We this will lead to improved data acquisition and better reporting guidelines during epidemic response.

While we acknowledge the significance of such a framework, we recognize a few limitations. One, despite our efforts, the Global.health core schema may not be comprehensive with respect to all pathogen groups and cannot ensure fidelity of the data collected. Second, currently the schema is in English, which may challenge its accessibility for non-native speakers. Third, depending on the dynamics and nature of an epidemic, national or governmental policies may not always support external data collection products. barriers may and evolving data formats might occur as the epidemic develops, which could impact the scale and quality of the data. For example, the first hundred cases might have detailed information (or vice versa, depending on the response team’s readiness), but less available with increasing number of cases ^32^. Or, as the epidemic grows, public health agencies might discontinue reporting individual-level case data and instead switch to reporting total numbers (or estimates thereof) of confirmed or suspected cases ^32^. For this, we may consider developing other augmented data ingestion frameworks.

It is also worth noting the initial challenge users might initially encounter adapting to datasets containing coded information (SNOMED, LOINC, etc.) rather than the usual simple database coding and text data format. These limitations notwithstanding, the updated Global.health schema has already been deployed in collaboration with national authorities, e.g., for Mpox data ingestion in the Democratic Republic of Congo, demonstrating practical feasibility in field settings. The digitization of WHO T0/T1 toolkits has also benefited the use of standardized data schema. We hope our work will encourage relevant and directed data capture across the infectious disease research community, ensuring standardized and efficient data collection for timely and informative decisions around appropriate public health responses.

## Ethics and consent

Ethical approval and consent were not required.

## Data Availability

No underlying data were associated with this study. OSF: Unified framework for the ingestion of early epidemic data for downstream data analytics. Dataset DOI 10.17605/OSF.IO/AKQ53 ^33^ This project contains the following extended data: Supplementary Table 1. Table of assessment and rating of the likelihood of data availability. Data are available under the terms of the Creative Commons Zero CC0. “No rights reserved” data waiver (CC0 1.0 Public domain dedication, Universal).
