# Unified framework for the ingestion of early epidemic data for downstream data analytics

**DOI:** 10.12688/wellcomeopenres.24776.2

**Published:** 2026-05-08

**Authors:** Everlyn Kamau, Sadie Kelly, Dhruv Darji, Amrish Y. Baidjoe, John S. Brownstein, Finlay Campbell, Abhishek Dasgupta, Marie-Amélie Degail, Anastasiia Demidova, Luca Ferretti, Aimee Han, Stephen Leshan Koyie, Patricia Ndumbi Ngamala, Olivier Le Polain, Amanda Rojek, Jacquelin Sauer, Samuel V. Scarpino, Kara Sewalk, Juliana Sopko, Stanislaw Zakrzewski, Laura Merson, Moritz U. G. Kraemer

**Affiliations:** 1 Francis I. Proctor Foundation, University of California San Francisco, San Francisco, California, USA; 2 Department of Biology, University of Oxford, Oxford, England, UK; 3 Nuffield Department of Medicine, University of Oxford, Oxford, England, UK; 4 Pandemic Sciences Institute, University of Oxford, Oxford, England, UK; 5 Medecins Sans Frontieres, Operational Centre Brussels (OCB), Brussels, Belgium; 6 Médecins Sans Frontières, Luxembourg Operational Research Unit (LuxOR), Luxembourg, Luxembourg; 7 Computational Epidemiology Lab, Boston Children’s Hospital, Boston, USA; 8 Harvard Medical School, Boston, Massachusetts, USA; 9 WHO Hub for Pandemic and Epidemic Intelligence, Berlin, Germany; 10 Research Software Engineering Group, University of Oxford, Oxford, England, UK; 11 World Health Organization, Geneva, Switzerland; 12 Independent Researcher, Limassol, Cyprus; 13 Department of Community Health Sciences, Boston University School of Public Health, Boston, Massachusetts, USA; 14 Institute for Experiential AI, Northeastern University, Boston, Massachusetts, USA; 15 Department of Public Health and Health Sciences, Northeastern University, Boston, USA; 16 Santa Fe Institute, Santa Fe, New Mexico, USA; 17 Technical University of Lodz, Lodz, Poland; 18 Institut Pasteur de Dakar, Dakar, Senegal

**Keywords:** Epidemic, Outbreak, Data Schema, Toolkits, Reporting Guidelines

## Abstract

**Background:**

Early-phase data during an epidemic are often heterogeneous and difficult to integrate across systems, therefore a need for standard tools and reporting guidelines to facilitate timely and reliable data collection. The Global.health team have developed a data schema for the ingestion of epidemic data, allowing interoperability where data curated to this schema are readily ingested into existing systems for analysis. This paper describes the definition of ‘core data’ within the Global.health schema to focus data collection on the most relevant and available data to inform epidemic response during the first 100 days of an outbreak.

**Methods:**

We used expert consultation and a structured literature review to identify key epidemiological questions and parameters that must be addressed during the first 100 days of an outbreak. Relevant digital toolkits and reporting frameworks were reviewed, and minimum data variables required for parameter estimation were identified. These variables were mapped to the existing Global.health schema and assessed for availability in early outbreak data from four recent epidemics. Variables were categorized by availability and those with sufficient early availability were retained in a proposed core schema. Data formats were harmonized with WHO Epi Core, T0 and T1 toolkits to enhance interoperability. A complementary modular schema was defined to capture pathogen-specific variables.

**Results:**

The literature review yielded 78 key epidemiological parameters relevant to early outbreak assessment, organized into eleven categories. Analysis of variable availability in early outbreak datasets showed that 42 of 140 variables in the existing Global.health schema were consistently available and suitable for inclusion in a core early-epidemic schema. Variables related to demographics, case status, symptom reporting, confirmation dates, outcomes, and exposure history were frequently available, while vaccination history, detailed treatment data, and certain clinical variables were less consistently reported. The resulting core schema comprises 42 interoperable variables across seven domains and aligns with WHO data standards and controlled terminologies.

**Conclusions:**

Standardized, interoperable data capture during the early phase of epidemics is essential to enable timely estimation of key epidemiological parameters and to inform response strategies. The Global.health core schema provides a minimum, evidence-informed dataset for early outbreak investigation while maintaining compatibility with WHO reporting standards. By prioritizing variables that are both epidemiologically critical and realistically available in early data streams, this framework supports improved data harmonization, analysis, and decision-making during the first 100 days of an epidemic.

## Introduction

The first weeks of an epidemic are crucial for mounting an effective response given the potential health and socioeconomic impacts of epidemics. ^1^ During this period, it is essential to obtain reliable estimates of key epidemiological parameters such as transmission rates and disease severity, both for known and newly emerging pathogens. This is also a critical time for official reporting systems to assess availability of response resources, evaluate clinical impact, plan rapid testing and interventions (including assessing their effectiveness), monitor disease progression and estimate potential burden on health care systems and society. However, in the early stages of an epidemic, data on infectious disease cases and associated metadata – such as case and death counts, locations, sources of infection, laboratory tests performed, and vaccination status – are often sparse and heterogeneously captured, thus hindering data integration, analysis and interpretation.

These limitations hinder the utility of available data in public health decision making, despite the importance of the information they hold. ^2^ Heterogeneity can result from variation in content, quality, volume, format, veracity, completeness, definition and management processes. ^3^ Additionally, a lack of standardization in data capture systems delays data integration for research and statistical analysis, which impedes understanding and forecasting of the trajectory of an epidemic. ^4^ This limits real-time use of data to effectively assess and optimize public health operations during epidemic response. ^5^

Another key issue in understanding disease dynamics is data access, which is limited by privacy concerns, legal and regulatory restrictions, conflicts of interest, complex processes to arrange data sharing agreements between entities, and the absence of trust, particularly in the digital era. ^2^ ^,^ ^6^ Socio-technical factors such as language barriers, differences between regional and national structures and rules that govern the dissemination of health information, imbalances in technical capacities, and power dynamics also impact the establishment of effective and meaningful data sharing. ^7^

The Global.health platform assembles and curates open-access emerging infectious disease data to support situational awareness and risk assessments for decision-makers, researchers and the public. ^8^ Global.health aims to make data sharing more efficient and data more openly accessible to communities and groups contributing to epidemic response. A central step towards this aim is data interoperability, which enables seamless access, exchange, integration, and coordinated use of data across different systems, applications, and organizations, while preserving meaning, integrity, and usefulness. Global.health advances interoperability through the development and implementation of a standardized data schema, designed to ensure that epidemiological data are collected or curated in a uniform format that supports rapid analysis and estimation of key epidemiological parameters. This schema, known as the Global.health Day 0 schema (available at https://github.com/globaldothealth/outbreak-schema ) has been applied to standardize epidemiological data from six previous outbreaks ( https://global.health). Its implementation has exposed a critical gap in the continuum of data available during epidemics, which is most pronounced in the early phase of an outbreak – defined as the first 100 days – when timely information is essential and the opportunity for containment is greatest. We hypothesize that extending and refining the Day 0 schema to better capture data relevant to early outbreak assessment and response will strengthen its utility as a tool for supporting decision-making during the initial stages of epidemics.

The purpose of the work described in this paper was to define the content of an early epidemic data schema to support timely, interoperable data collection and analysis during the initial phase of an outbreak. Central to the development of such a schema is the identification of key questions and parameters that are most important to estimate early during an epidemic. Here, we describe how these questions and parameters were determined through background research, literature review and expert interviews, and how they informed the development of the Global.health early epidemic data schema. We also describe the alignment of the Global.health data schema ^9^ with existing World Health Organization (WHO) toolkits (T0 and T1 forms) and define a minimum set of data required for epidemic investigation, particularly during the early phase (first 100 days) of an infectious disease outbreak. Ultimately, this work aims to ensure that epidemiological data curated to the schema can be shared in a standardized format that is readily ingested by existing and future analytical systems for epidemic analytics.

## Methods

**Figure 1.**
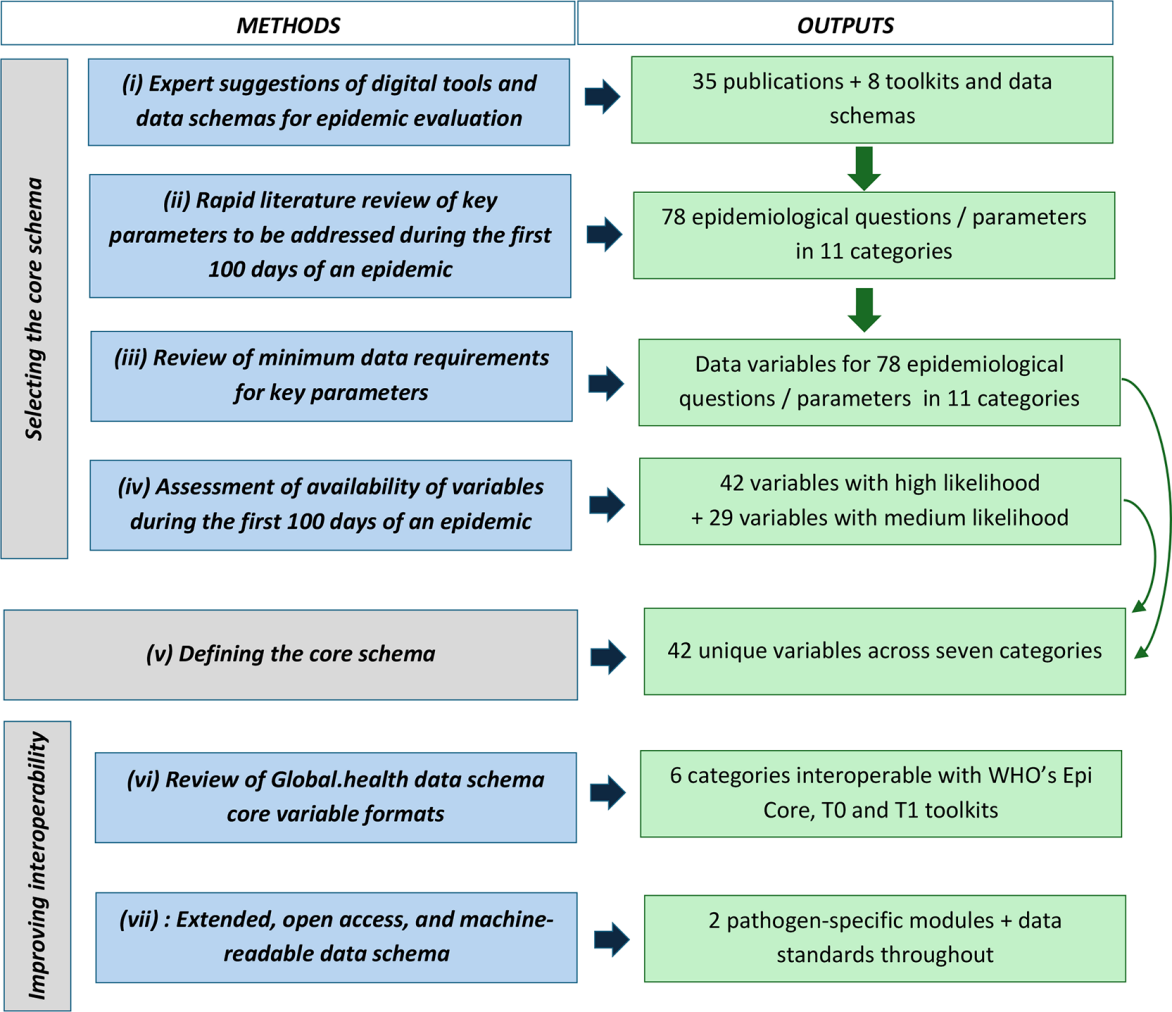
Conceptual framework of this study. Schematic illustrating the analyses and steps involved.

Our workflow and qualitative analysis were carried in multiple steps which are illustrated in Figure 1 and are described as follows:

***(i) Selecting the core schema: Expert suggestions of digital tools and data schemas for epidemic evaluation.*** We first invited experts to identify existing toolkits and data schemas for epidemic data collection. A group of 22 scientists and academic researchers engaged in disease modelling, epidemiologists in national and international public health agencies (including three WHO offices) and outbreak specialists at humanitarian organizations were invited to share titles and links to relevant resources. Their suggestions included tools in the peer reviewed literature as well as additional resources from the grey literature.

***(ii) Selecting the core schema: Rapid literature review of key parameters to be addressed during the first 100 days of an epidemic.*** Next, we conducted a literature review to identify key epidemiological parameters or questions that must be addressed during the first 100 days of an infectious disease epidemic. We searched the PubMed database was searched using the following terms: (((((epidemiology) OR (epidemiological)) AND ((((((outbreak) OR (outbreaks)) OR (disease outbreak)) OR (epidemic)) OR (epidemics)) OR (pandemic*))) AND ((((parameter*) OR (question*)) OR (data)) OR (information))) AND (((early) OR (initial)) OR (priority))) AND ((((((((((incubation period) OR (case fatality rate)) OR (case fatality rate (CFR))) OR (risk factors)) OR (basic reproduction number)) OR (R _0_)) OR (effective reproduction number)) OR (serial interval)) OR (delay distributions)) OR (generation time)). The search was conducted on 23 ^rd^ June 2024. The first 200 English language articles ranked by PubMed as most relevant to the search terms were combined with the resources identified through expert suggestion and deduplicated. The titles of all content were reviewed by two independent reviewers to identify those relevant to early outbreak response. Discrepant choices were resolved via discussion between the two reviewers. The full text of each selected title was reviewed to identify those with relevant data questions and parameters. Those selected by at least one reviewer were used for data extraction. Extracted content was categorized into types of parameters.

***(iii) Selecting the core schema: Review of minimum data requirements for key parameters**.* The categorized parameters and questions extracted from the toolkits and literature in (i) and (ii) above were evaluated to identify the data variables required for their calculation or estimation. Variables were listed per parameter, then grouped using categories defined in prior work by Perrocheau et al. ^21^ Ten of the 22 individuals who participated in the expert suggestions provided iterative review of the resulting list to agree on the completeness and suitability of the variables and categories for addressing questions during the early phase of a public health response. Iteration resulted in consensus on a draft list of variables. The list was reviewed against the data reporting requirements of the International Health Regulations (IHR, 2005) to ensure that the variables covered the IHR reporting requirements.

***(iv) Selecting the core schema: Assessment of availability of variables during the first 100 days of an epidemic.*** Variables defined in (iii) were identified, were available, within the Global.health day 0 schema and assessed for availability based on their presence or absence from previous Global.health data sources ^8^ during the first 100 days of an epidemic. Availability was determined as the count or frequency of records containing a variable of interest within 100 days of the first identified case. Data from epidemics including Ebola (2018–2020), Marburg (2024), COVID-19 (2019–2023) and Mpox (2023–2024) were assessed. Each variable was assigned a rating of ‘high’, ‘medium’, or ‘low’, indicating the mean availability of the variable in early epidemic data as ≥80%, ≥50% to <80% and < 50%, respectively. Those with low availability were excluded from the early outbreak variable selection.

***(v) Defining the core schema.*** The outputs of (iii) and (iv) above were used to define a subset of the Global.health data schema as a ‘core’ schema for early outbreak assessment and response. First, the Global.health variables that matched the minimum data variables for key parameters were selected. This list was then reduced to include only those variables evaluated as high or medium availability in early epidemic data.

***(vi) Improving interoperability: Review of Global.health data schema core variable formats.*** We compared the format of the variables included in the newly defined Global.health core data schema with the formats used in three of the key existing toolkits identified amongst the expert suggestions. Global.health formats were adjusted to align with WHO’s Epi Core, T0 and T1 formats to ensure that data will be interoperable across these tools.

***(vii) Improving interoperability: Extended, open access, and machine-readable data schema***. Additional modular schemas were subsequently defined using the WHO T0/T1 and disease-specific toolkits identified in (i) above. The additional variables were selected to capture pathogen specific detail that were missing from the core schema. This group of variables were defined as a ‘modular’ schema and includes variables related to exposure, treatments and vaccination. The format of all data variables was reviewed against the data standards in use by WHO. Appropriate controlled terminologies were applied as relevant.

## Results

### Data schemas for epidemic evaluation

Experts identified 35 publications as relevant to the project aims and eight unique toolkits and data schemas (see Table 1). Six of the eight resources were designed to ingest data from epidemic field investigations.

**Table 1.** Toolkits. Summary of existing toolkits and digital resources for infectious disease data and information collection (WHO: World Health Organization; CDC: Centers for Disease Control).

Digital tool	Brief description of purpose, source and design
WHO T0 case investigation form ^19^	The initial generic form designed to help outbreak field investigators rapidly understand an epidemic and propose initial control measures. It supports the collection of the minimum Epi Core ^20^ variables for epidemic investigation, ^21^ and was designed to help describe the epidemic over time, geographical spread and persons affected to guide decisions regarding the first measures to control the epidemic. Data collected from the WHO T0 case investigation form ^19^ can also be used to generate hypotheses about the case, source, and transmission mode of a pathogen. The additional variables collected on the T0 form outside of the specified Epi Core variables were collated from the most common variables indicated on the WHO Disease outbreak toolkits, ^22^ which were created to enable rapid specification of a case definition, collection of appropriate data for the disease and for field workers to have a resource for tools and training.
WHO T1 case investigation form for outbreaks of unknown cause ^19^	Used to collect detailed information on any situation involving an epidemic of unknown origin. It is designed for initial data collection to identify the epidemic source, mode of transmission or agent involved, and is composed of 462 variables organized in five categories, many of which are conditional or symptom-based checklists. The clinical variables in the WHO T1 form are grouped per functional body system (e.g., neurological, digestive, cutaneous), which is preferred as opposed to the syndromic approach when the disease is unidentified. ^19^ The exposure variables in the WHO T1 form support the investigation of risks related to transmission, food or water contamination with pathogens and environmental toxins and hazards (e.g., heavy metals) that can lead to an outbreak.
The European Surveillance System (TESSy)	The TESSy portal facilitates collection, analysis, and dissemination of indicator- and event-based surveillance data in infectious disease and associated health issues. The TESSy metadata set lists the collected data for a wide range of pathogens, from both aggregate and case-based data. The European Surveillance System (TESSy), is now integrated into EpiPulse with the five Epidemic Intelligence Information System (EPIS) platforms and the Threat Tracking Tool (TTT). ^23^ ^–^ ^26^ TESSy’s standardised data formats and validation rules are provided within the metadata to improve data quality, but the rationale for the collection of each variable is not provided. Data sources include those that are open access such as FluTrackers, monitoring websites and trusted media sites, as well as WHO sources and other access-restricted sources. Furthermore, access to EpiPulse is restricted.
The article ‘Key data for outbreak evaluation: building on the Ebola experience’ ^27^	This draws from the authors prior experiences in outbreak data collection and analysis and provides a checklist of data needed to quantify severity and transmissibility, characterize heterogeneities in transmission and their determinants, and assess the effectiveness of different interventions. The article recommends that data are differentiated into individual-level data, exposure data, and population-level data, and the checklist also highlights the potential issues and biases that can be present in these data. The setting for data capture in the article is also primarily field investigations, like the WHO toolkits.
WHO PISA (Pandemic Influenza Severity Assessment)	PISA details the requirements for influenza surveillance and how to assess the impact of a potential outbreak. ^28^ It focuses on assessment of (i) transmissibility using case incidence and positive test rates, (ii) severity using deaths and hospitalizations, and (iii) impact using incidence, excess pneumonia/influenza or all-cause mortality, confirmed cases, hospital admissions, absenteeism in school and workplaces, and healthcare resources use such as beds occupied.
CDC framework for assessing epidemiological effects of influenza epidemics and pandemics ^29^	Similar to the WHO’s PISA risk assessment on transmissibility and severity. Transmissibility is assessed using R _0_, the serial interval and the attack rate, while severity is assessed from case fatality, hospitalization records and genetic markers of virulence. This document also lists the strengths and limitations of each parameter and provides considerations towards evaluation of data quality. The WHO PISA suggests that parameters should be reviewed by age groups, and severity should consider presence or absence of underlying chronic diseases.
Key indicators for WHO priority pathogens with a confirmed outbreak	WHO’s key epidemiological indicators and recommended analyses for surveillance of priority pathogens with a confirmed outbreak have been published for various diseases including cholera, ^30^ measles, yellow fever and meningitis. ^31^ ^,^ ^32^ They focus on incidence, attack rate, severity (case fatality) and test positivity. Only for cholera have the indicators been linked to specific data points and rationale provided.
Global.health day 0 core schema	The Global.health day 0 core schema ^9^ uses a common data model to standardize key epidemiological epidemic data that includes details for case status, location, age, sex, symptoms, transmission, hospitalization, treatment, vaccination, outcome, contacts, travel history, occupation, genomics and more. Each individual case is assigned a unique identification number when added to a line list and data are de-identified to protect individual privacy. The Global.health data sources are primarily those that are publicly available. Originally designed in the context of COVID-19 data curation ^33^ and most recently updated for mpox reporting (covering the 2022 global epidemic) where it was based heavily on the WHO mpox case report form. It collects data and metadata from official and non-official online public sources and provides the original source(s) of information for each case to support transparency and data sharing. These variables are specified by minimum case requirements and are pathogen-agnostic.

### Key parameters to be addressed during the first 100 days of an epidemic

**Figure 2.**
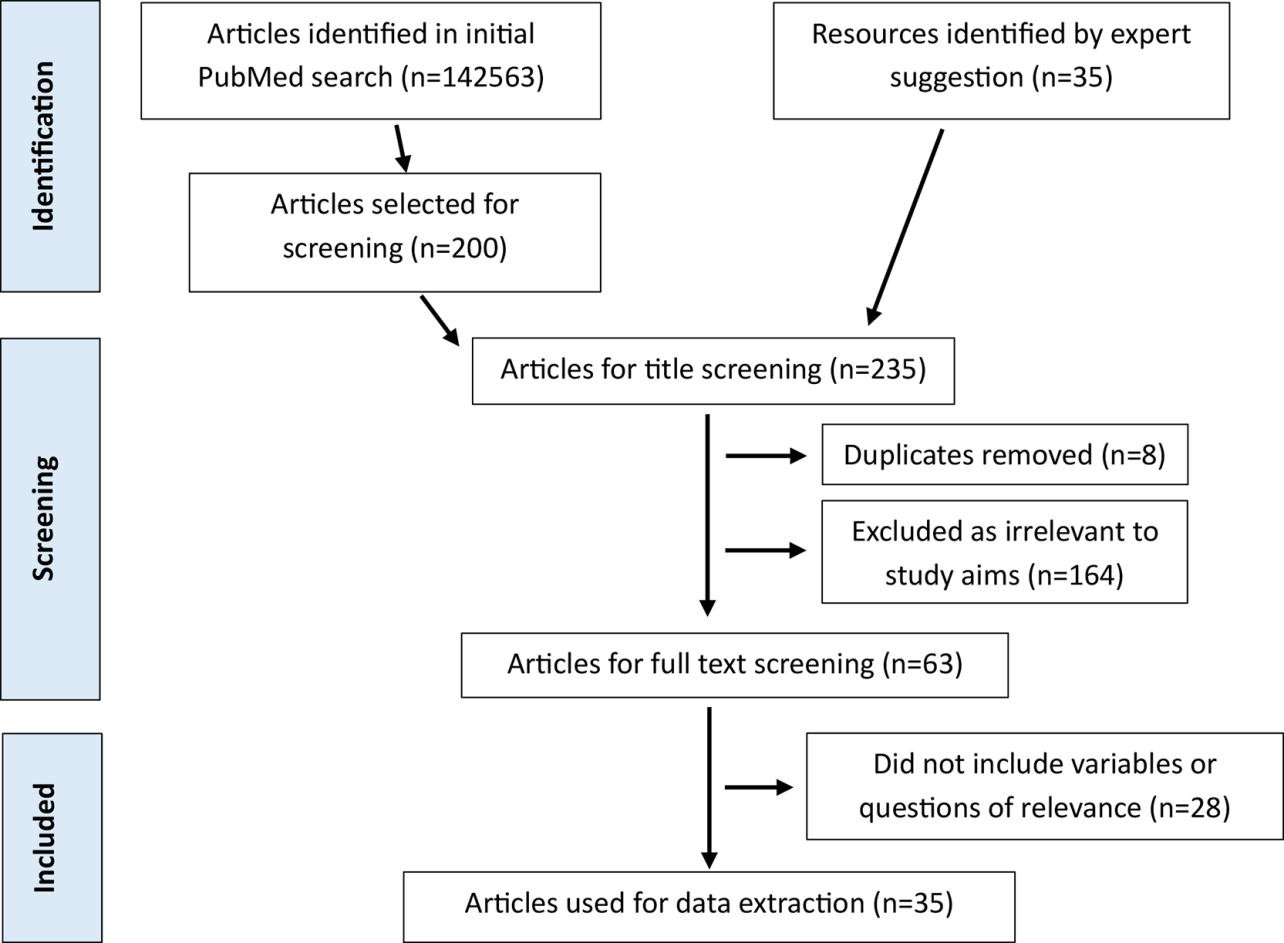
Literature search (PRISMA diagram of scoping review). Literature search workflow to identify and extract key questions and parameters analyzed to guide public health response during the early stages of an epidemic or outbreak.

Our search terms identified 142,563 potentially relevant articles. The 200 titles ranked by PubMed as most relevant were combined with the 35 expert identified publications. Following removal of duplicates, the titles and abstracts from the remaining 235 were screened to identify 63 of potential relevance to the study. Full text review of these resources resulted in data extraction from 35 found to contain parameters relevant to understanding the first 100 days of an outbreak ( Figure 2). We identified and extracted 78 unique epidemiological questions or parameters from the selected literature. Parameters were categorized into eleven categories as described in Table 2.

**Table 2.** Key categories and parameters. A summary of categories and parameters or variables considered important for infectious disease epidemic investigation.

Category	Description and some of the parameters included
Epidemiological parameters	Used to model transmission and predict epidemic growth rate. Include basic reproduction number, incidence rate, effective reproduction number, incubation period, serial interval, generation time and secondary attack rate.
Delay distributions	Describe delays between events in the infection history and reporting, which affect transmission and can have implications for epidemic control.
Disease severity factors	Include case fatality rate, hospital and intensive care unit admission rates.
Risk factors for epidemic growth, susceptibility to infection and poor outcomes	Include individual level demographic factors, medical factors (e.g., pre-existing conditions, vaccination history, reinfections), occupational factors (e.g., health care worker status or other risks from specified occupations depending on the pathogen), behavioral factors (e.g., details of exposure and travel history to determine settings and risk factors of transmission).
Where/Who factors	Important for inferring transmission heterogeneities or risk of infection across different locations or population groups.
Clinical features	Case defining or prognostic features, laboratory results and vital signs.
Contextual and external factors	Broader population-level indicators for surveillance bias (how an infected case was identified to the healthcare system), impact of human mobility patterns, climate and other drivers of transmission, as well as knowledge of pathogen genomic lineage for detailed analyses.
Diagnostic factors	Knowledge of testing methods and strategies across different locations to interpret incidence and other epidemiological aspects listed above.
Surveillance parameters	High-level estimates of the proportion of infections reported, asymptomatic cases as well as peak-time forecasts of incidence, useful to model the impact of the epidemic. These could be a function of, or derived from, the contextual and external factors.
Impact of pharmaceutical interventions	Medical treatments and vaccinations, and their impact on incidence and individual outcomes.
Non-pharmaceutical interventions	Non-pharmaceutical interventions, or “Public Health and Social Measures”, ^34^ such as isolation procedures, and other behavioral interventions (e.g. hand-washing, mask-wearing) and their impact on incidence and individual outcomes.

### Minimum data reporting requirements for key parameters

Data variables identified by the project team and expert reviewers for each key parameter and question are listed in Table 2. Variables included in the table were found to cover all IHR reporting requirements. The final variables were considered those important to early epidemic investigation.

### Availability of variables during the first 100 days of an epidemic

Of the current 140 variables in the Global.health data schema, 42 had a high likelihood of being captured in a line list in early epidemic data ( **Supplementary Table S1**). These variables included parameters that describe pathogen name, case status, location of the case report, list of symptoms, date of case report, outcome, date of case confirmation, date of outcome evaluation, occupation, where contact with infected or suspected cases occurred, travel history and sources of information. Where travel history is mentioned, the travel location is indicated.

There were 29 variables with a medium likelihood of availability in epidemic data sources including pre-existing conditions, vaccination information, date of symptom onset, pathogen strain subtype, diagnostic test, contact with animal or insect bites, date of entry into the country, type of medical treatment and exposure to water or chemical agents ( **Supplementary Table S1**). Other variables (n = 56) were rated as low likelihood of availability. These included confirmed prior infection, co-infection with other pathogens, number of vaccine doses, date of first vaccination, admission to intensive care unit, date of isolation, vital measurement results, dates of medical treatment, type of specimen and mode of travel.

Evaluation of the Global.health data also had a low availability of data on whether an individual visited a healthcare facility several days prior to symptom onset, or how a case was found (which might help to assess surveillance bias), as well as reliably tell the date of the healthcare visit. The reports cannot reliably differentiate between primary and secondary cases within contact exposure, but where death and treatment are mentioned, the date of death and type of treatment are often indicated. Other things to note: (i) vaccination information is available depending on the causative pathogen and hence the low likelihood of availability as noted above, (ii) ‘death’ is the most commonly reported outcome while recovery or date of recovery is not always mentioned, (iii) where symptoms may not be mentioned, an asymptomatic case might be assumed, (iv) home monitoring may not be distinguished from the general ‘isolation’ variable, and (v) pre-existing conditions are reported more frequently than previous infections. The current Global.health schema variables on mass gatherings were specific to COVID-19 and therefore unlikely to be available for other epidemics.

### The core schema

Selection of the data variables for key parameters and reduction of this list to those with high or medium availability resulted in a list of 42 unique variables across seven categories. These variables, featured in Table 3, comprise the Global.health core schema.

**Table 3.** **Minimum data requirements**. This table highlights variables or information that would be considered the minimum requirements for various categories and parameters.

Category	Parameter(s)	Minimum variables or information required
Epidemiological parameters	Incubation period ^a^	Date of symptom onset Exposure date or date of last contact to the suspected source (e.g., animal, infectious individual, toxin, etc.) Travel dates (e.g., exposure interval at the source and symptom onset in other location)
	Basic reproduction number (R0) ^b^ Effective reproduction number (Rt) ^c^	Date of symptom onset of confirmed cases Date of case confirmation (Additional information on vaccination is recommended for *Rt* where suitable)
	Incidence rate ^d^, attack rate ^e^, growth rate ^f^	Case status (e.g., confirmed, probable, suspected) Case location Date of case report
	Secondary attack rate	Date of symptom onset in *infector* Date of symptom onset in *infected*
	Serial interval distribution ^g^	Household size (Other supplementary data elements include travel history location(s) and setting where contact occurred with the confirmed/suspected case)
Delay distributions	Measures of delays between events in the infection history and reporting (e.g., Date of symptom onset to date of reporting)	Date of symptom onset Date of case report Date of isolation Date of hospitalization Date of hospital discharge Outcome (e.g., hospitalization, death, recovery) Date of outcome evaluation Date of specimen collection Date of case confirmation
Disease severity factors	Measure of severity e.g., Infection fatality ratio/Infection fatality rate, case-hospitalization ratio	Case status (e.g., confirmed, probable, suspected) Disease outcome (hospitalization, death, recovery) Date of outcome evaluation Age Race and/or ethnicity Clinical vulnerability Occupation (e.g., healthcare worker) Date of case report Intensive care treatment
Risk factors for epidemic growth, susceptibility to infection and individual outcomes	Demographic, medical and occupation risk factors	Travel history location(s) Date of country entry Date of travel start Date of symptom onset Age Sex at birth Date of hospitalization Pre-existing conditions including pregnancy Presenting symptoms Presenting treatments Vaccination (including timing) Occupation (e.g., healthcare worker) Contact status with infected or suspected case Close proximity with animals or insect bites prior to symptom onset Exposure to harmful water or chemicals Visiting a healthcare facility days prior to symptom onset Infection history (previous similar infection)
Diagnostic factors	Diagnostic performance	Diagnostic test Results from negative and positive control samples Type of specimen
Impact of pharmaceutical interventions	Impact on incidence and individual outcome	Medical treatments (including timing) Vaccination (including timing) Outcome (e.g., hospitalization, death, recovery)
Impact of non-pharmaceutical interventions	Impact on incidence and individual outcome	Type of interventions and date Date of symptom onset Date of isolation Outcome (e.g., hospitalization, death, recovery) Time series of suspected, probable or confirmed cases (population level)

^a^ The interval between exposure and initial occurrence of signs and symptoms.

^b^ The expected number of cases generated by a single case in a population where all individuals are susceptible to infection.

^c^ The number of cases generated in the current state of a population.

^d^ The number of new cases in a population within a specified period of time.

^e^ The proportion of an at-risk population that contracts the disease during a specified time interval.

^f^ How quickly the numbers of infections are changing over a period of time.

^g^ The interval between symptom onset in an index case and in a secondary case.

### Improving interoperability: Review of Global.health data schema core variable formats

The structure of the core schema was aligned with the structure of the WHO’s Epi Core, T0 and T1 toolkits, ensuring that variables in both systems are aligned and data are captured similarly. The categories were structured as follows:

I. *Demographics*: includes variables with responses such as age, gender, sex at birth, case status (e.g., confirmed, suspected, probable), locality or residence location and job occupation. The residence query results in a location structure that is pre-filled by mapbox with latitude and longitude, ISO code for country and geographical administrative information.
II. *Medical History*: this category contains variables on pre-existing conditions including pregnancy, co-infections, history of the same infection prior to the current diagnosis and vaccination information.
III. *Clinical Presentation*: includes variables on reported symptoms, date of symptom onset, hospitalization, intensive care treatment, outcome of illness (e.g., death, recovered, post-acute sequelae), vital measurement results, pharmaceutical and non-pharmaceutical treatments.
IV. *Laboratory Information*: including genomic information, testing information (e.g., type of specimen, method of testing date of sample collection, pathogen strain/subtype) and date of case confirmation.
V. *Exposure*: contains variables indicating likely sources of exposure e.g., contact with suspected or confirmed cases or infected animals/animal products, or through environmental exposure (water sources, chemicals, exposure).
VI. *Source Information*: contains variables on data origin (e.g., government bulletins, ministry of health reports, media platforms, etc.), date of entry and/or modification and curator’s details and comments.

Several variable responses in the Global.health schema e.g., symptoms, specimen type, pre-existing medical conditions and type of medical treatment can be selected from predefined lists with an option for free text entry.

### Improving interoperability: Extended open access and machine-readable data schema

Based on the results above, we updated the Global.health ‘core’ schema to capture variables relevant to all epidemic types and added a ‘modular’ schema. The latter contains two modules with variables related to exposure and interventions e.g., treatments and vaccination, which can be adjusted to become pathogen specific ( Table 4). The variables in both schema setups were reviewed against existing toolkits in terms of data type, question text, and response options to ensure consistency and interoperability with other data capture specifications. The schemas specify syntactic and semantic standard codes for each variable according to the WHO SMART guidelines, ^10^ to allow for interoperability of shared data. These codes were prepared from standardised dictionaries including SNOMED-CT (International), ^11^ SNOMED-GPS (Global Patient Set), ^12^ LOINC, ^13^ ICD-11, ^14^ ICHI ^15^ and ICF, ^16^ and align with existing codes used in other WHO digital adaptation kits for interoperability within WHO systems. For each variable, multiple codes were specified where applicable to allow better interoperability among users or systems working with the resulting curated dataset. Collectively, the core and modular schema form a digital adaptation kit, which as part of the WHO SMART guidelines can be adapted to contain additional variables capturing exposure types, treatments, and vaccinations.

**Table 4.** Exposure and intervention modules. Each column represents the two Global.health schema modules. In each column we show the representative data elements and examples of specific variables related to these elements. Currently, the intervention module has only three data elements.

Exposure module	Intervention module
Contact with case (e.g., contact ID, contact setting, date of last contact)	Pharmaceutical interventions – vaccinations (e.g., number of doses, vaccine name, vaccination date, side effects)
Mass gathering (e.g., mass type, date of event, location of event)	Pharmaceutical interventions – treatments (e.g., type and name of treatment, route, start and end date, daily dose, traditional treatment)
Animal contact (e.g., animal species, date and location of contact with animal, insect bites or stings, contact with skinned wild game, raw animal meat of blood)	Non-pharmaceutical interventions (e.g., face mask, social distancing, hand washing, school closure)
Travel history (e.g., date of entry into country, location of travel, mode of travel)	
Treatment (e.g., visit to healthcare facility, type and location of facility, visit to a traditional healer)	
Water source (e.g., type of drinking water source, contact with flood water)	
Chemical source (e.g., potential source of chemical exposure, place and duration of exposure, suspected chemical product)	

## Discussion

Infectious disease surveillance requires timely and reliable data ingestion processes which are often complex and fragmented across institutions and governmental entities. Analytical methods, tools and resources for epidemic investigation have grown significantly over the last two decades, however, data acquisition and interoperability have remained a challenge and are difficult to standardize across pathogens and diseases. We have developed a unified data ingestion framework for descriptive mapping of variables collected during an epidemic response, which has a wide scope of parameters and variables preset with a uniform coding system for optimal interoperability. We examined the data collection variables in the Global.health data schema to assess interoperability with WHO systems and ensure that the output of data curated to the Global.health schema can be shared in a standard format readily ingested to support key epidemiological tasks identified by the WHO.

Currently, the occupational risk factors in the Global.health schema will differ depending on the type of epidemic, but it would be desirable to have agnostic occupation categories applicable to all epidemics. In our assessment of data availability for the minimum reporting requirements, we did not evaluate the likelihood of availability by geographical origin. This would be useful to identify gaps in data collection methods or surveillance system structures that could potentially challenge harmonizing surveillance data across countries.

The low availability of some data variables is likely to impact epidemic and outbreak response and epidemiological parameter estimation. For instance, inadequate information on vaccination might hinder evaluation of coverage and impact immunization services on disease burden. Similarly, inadequate information on hospital discharge could hinder predictive models for estimating the discharge probability of acute care patients. We found that the date of specimen collection was lacking, which would also impact understanding of when a disease process was present in a patient. The ongoing efforts for improved data collection should emphasize such variables, and the several others as they play an important role in monitoring disease response and management.

In future work, we will further assess whether the key parameters identified here can be adapted to the field resources available during an epidemic investigation through a user survey, the outcome of which will be used to update the core Global.health schema and the minimum data requirements for epidemic evaluation. This will lead to improved data acquisition and better reporting guidelines during epidemic response. We also acknowledge that data representation might vary by location and that future work should focus on developing specific guidelines by region for improved early data availability and accessibility.

While we acknowledge the significance of such a framework, we recognize its limitations. Firstly, despite our efforts, the Global.health core schema may not be comprehensive with respect to all pathogen groups as ‘Disease X’ may present with unrepresented risk factors or clinical characteristics. Second, the schema cannot ensure fidelity of the data collected. Third, currently the schema is in English, which may challenge its accessibility for non-native speakers. Fourth, depending on the dynamics and nature of an epidemic, national or governmental policies may not always support external data collection products. Barriers may and evolving data formats might occur as the epidemic develops, which could impact the scale and quality of the data. For example, the first hundred cases might have detailed information (or vice versa, depending on the response team’s readiness), but less available with increasing number of cases. ^17^ Or, as the epidemic grows, public health agencies might discontinue reporting individual-level case data and instead switch to reporting total numbers (or estimates thereof ) of confirmed or suspected cases. ^17^ For this, we may consider developing other augmented data ingestion frameworks.

It is also worth noting the initial challenge users might initially encounter adapting to datasets containing coded information (SNOMED, LOINC, etc.) rather than the usual simple database coding and text data format. These limitations notwithstanding, the updated Global.health schema has already been used in collaboration with national and international health authorities during public health emergencies, demonstrating practical feasibility in field settings. The digitization of WHO T0/T1 toolkits has also benefited the use of standardized data schema. We hope our work will encourage relevant and directed data capture across the infectious disease research community, ensuring standardized and efficient data collection for timely and informative decisions around appropriate public health responses.

### Ethics and consent

Ethical approval and consent were not required.

## Data availability

No underlying data were associated with this study.

### Extended data

OSF: Unified framework for the ingestion of early epidemic data for downstream data analytics. Dataset. DOI 10.17605/OSF.IO/AKQ53 ^18^

This project contains the following extended data:

• Supplementary Table 1 Table of assessment and rating of the likelihood of data availability.

Data are available under the terms of the Creative Commons Zero CC0. “No rights reserved” data waiver (CC0 1.0 Public domain dedication, Universal).
