# Supplementary material for: Unified framework for the ingestion of early epidemic data for downstream data analytics
Source: Wellcome Open Res. 2026 May 8;10:524. Originally published 2025 Sep 22. [Version 2] doi: 10.12688/wellcomeopenres.24776.2 (PMC13247574; doi:10.12688/wellcomeopenres.24776.2)
Supplement: Peer review history

# Reviewer report: Reviewer response for version 2

**DOI:** 10.21956/wellcomeopenres.29012.r155933

**Published:** 2026-06-08

**Authors:** Lilia Perfeito

**Affiliations:** 1 LIP–Laboratory for Instrumentation and Experimental Particle Physics, Lisboa, Portugal

I was happy to review this paper as it addresses a key problem in epidemiology, particularly in new outbreaks, which is the difficulty in finding standardized data across multiple countries. Global.Health seems like a promising tool to do that, if it is adopted by health authorities.

 I believe the clarifications made after version 1 have made the paper stronger and I do not have any other major comments; just 3 minor ones:

 1 - If possible, it would be helpful for the community if the authors made available the 78 key parameters that they identified in the literature (as a supplementary table). Since only 42 were included, I am curious about what we may be missing out and whether in the future those may be included.

 2 - In figure 2, the PRISMA diagram, the authors start with 35 expert-curated resources and end up with 35 articles used for data extraction. Is this just a coincidence or were these the same? 

 3 - in page 6 of the pdf, we can find the following excerpt:

 “(iv) Selecting the core schema: Assessment of availability of variables during the first 100 days of an epidemic. Variables defined in (iii) were identified, were available, within the Global.health day 0 schema and assessed for availability based on”

 Should the second “were” be “where”?

Is the work clearly and accurately presented and does it cite the current literature?

Yes

If applicable, is the statistical analysis and its interpretation appropriate?

Not applicable

Are all the source data underlying the results available to ensure full reproducibility?

Yes

Is the study design appropriate and is the work technically sound?

Yes

Are the conclusions drawn adequately supported by the results?

Yes

Are sufficient details of methods and analysis provided to allow replication by others?

Yes

Reviewer Expertise:

Complex Systems

I confirm that I have read this submission and believe that I have an appropriate level of expertise to confirm that it is of an acceptable scientific standard.

# Reviewer report: Reviewer response for version 1

**DOI:** 10.21956/wellcomeopenres.27296.r140042

**Published:** 2026-01-13

**Authors:** Geneva Tamunobarafiri Igwama

**Affiliations:** 1 University of Akron, Akron, Ohio, USA

This manuscript discusses a framework for gathering and standardizing early epidemic data to assist with epidemiological analysis. The authors introduce the Global. health data schema, which aims to enhance the compatibility and usability of varied outbreak data. They evaluate the framework against World Health Organization (WHO) tools and conduct a literature review to identify key epidemiological parameters relevant in the initial 100 days of an epidemic, alongside expert consultations and reviews of data from recent epidemics like Ebola, COVID-19, Mpox, and Marburg. 

 The authors compile 78 essential epidemiological parameters into 11 categories and define the minimum data necessary to estimate these parameters. They refine the Global. health schema into a "core" schema and adaptable modular components. The paper emphasizes that better standardization and prioritization of early outbreak data can improve real-time data analysis and public health decisions. 

 The manuscript presents relevant insights into public health, aligning well with international guidelines and demonstrating solid methodology through a clear workflow. It offers a practical, openly available schema already in use in some field settings. However, there are concerns regarding the lack of detail in the expert consultation process, which may hinder reproducibility. The literature review could benefit from improved rigor, and availability assessments are noted to be somewhat subjective. 

 Major comments that require attention include improving transparency and rigor in expert selection, clarifying the literature review methods, and providing a validation example for the framework's practical benefits. Minor recommendations suggest addressing geographic disparities in data availability, discussing language accessibility, and improving the clarity of data tables. 

 The manuscript is a significant contribution to epidemic preparedness, but it needs major revisions for methodological transparency and validation before full acceptance.

Is the work clearly and accurately presented and does it cite the current literature?

Yes

If applicable, is the statistical analysis and its interpretation appropriate?

Yes

Are all the source data underlying the results available to ensure full reproducibility?

Yes

Is the study design appropriate and is the work technically sound?

Yes

Are the conclusions drawn adequately supported by the results?

Yes

Are sufficient details of methods and analysis provided to allow replication by others?

Yes

Reviewer Expertise:

Public health, digital health, Medicine, Nursing, Epidemology, Health,

I confirm that I have read this submission and believe that I have an appropriate level of expertise to confirm that it is of an acceptable scientific standard, however I have significant reservations, as outlined above.

## References

[1] : Unified framework for the ingestion of early epidemic data for downstream data analytics. *Wellcome Open Research* .2025;10: 10.12688/wellcomeopenres.24776.1 10.12688/wellcomeopenres.24776.1 39944230

# Response

**Published:** 2026-03-21

**Authors:** Everlyn Kamau

**Affiliations:** University of California San Francisco, San Francisco, California, USA

**Reviewer 2** This manuscript discusses a framework for gathering and standardizing early epidemic data to assist with epidemiological analysis. The authors introduce the Global. health data schema, which aims to enhance the compatibility and usability of varied outbreak data. They evaluate the framework against World Health Organization (WHO) tools and conduct a literature review to identify key epidemiological parameters relevant in the initial 100 days of an epidemic, alongside expert consultations and reviews of data from recent epidemics like Ebola, COVID-19, Mpox, and Marburg. 

 The authors compile 78 essential epidemiological parameters into 11 categories and define the minimum data necessary to estimate these parameters. They refine the Global. health schema into a "core" schema and adaptable modular components. The paper emphasizes that better standardization and prioritization of early outbreak data can improve real-time data analysis and public health decisions. 

 The manuscript presents relevant insights into public health, aligning well with international guidelines and demonstrating solid methodology through a clear workflow. It offers a practical, openly available schema already in use in some field settings. However, there are concerns regarding the lack of detail in the expert consultation process, which may hinder reproducibility. The literature review could benefit from improved rigor, and availability assessments are noted to be somewhat subjective. **Thank you. The entire methods section has been rewritten to add detail, improve reproducibility and insure clarity of process.**

 Major comments that require attention include improving transparency and rigor in expert selection, clarifying the literature review methods, and providing a validation example for the framework's practical benefits. Minor recommendations suggest addressing geographic disparities in data availability, discussing language accessibility, and improving the clarity of data tables.  T **hank you. Disparities in data availability: - This was not a focus of this paper. We were interested in assessing the likelihood of data variables being present in the Global.health data sources. While geographic disparities may play a role in data availability, addressing that is beyond the scope of this work. We have highlighted this in the discussion for future work. Language accessibility: - While the schema is currently in English, accessibility in other languages is possible but not within scope of our current work. Clarity of data tables: - Thank you for identifying this lack of clarity. We have rewritten the results section to match the rewritten methods section. Tables are linked to specific stages of results to improve clarity of process.**

 The manuscript is a significant contribution to epidemic preparedness, but it needs major revisions for methodological transparency and validation before full acceptance. **Thank you. We have added detail to the specific issues raised and look forward to further feedback.**

# Reviewer report: Reviewer response for version 1

**DOI:** 10.21956/wellcomeopenres.27296.r135848

**Published:** 2025-11-11

**Authors:** Juan Torres Munguía

**Affiliations:** 1 Georg-August-Universität Göttingen, Göttingen, Germany

Dear Editorial Team, dear authors,

 Thank you very much for the opportunity to review the paper entitled "Unified framework for the ingestion of early epidemic data for downstream data analytics." I would like to sincerely commend the authors for their effort and dedication. The topic addressed is highly relevant and impactful.

 Overall, the paper presents a proposal for the collection of data during the early phase of a disease outbreak by establishing a unified schema that captures the most relevant indicators for decision-making. I believe the manuscript is suitable for indexing after a medium revision. In particular, I have the following comments:

 Abstract

 1. The goal of the paper is not mentioned. Please add it in the background subsection.

 Methods

 2. The description of the methods could be improved. Briefly describe Figure 1, indicating that it represents a methodology based on different steps.

 3. The indication that the early phase refers to the first 100 days should be introduced earlier in the text, ideally at the first instance it is mentioned, to ensure clarity for the reader.

 Introduction

 4. Please add a paragraph about the impact of diseases and the relevance of studying them. For instance, include the number of deaths from COVID-19 using data from the WHO COVID-19 dashboard or the number of disease outbreaks worldwide from this paper: https://doi.org/10.1038/s41597-022-01797-2

 5. Define the concept of data interoperability.

 6. Add a paragraph describing the goal of the paper and its structure.

 Methods

 7. Figure 1 (Conceptual framework of this study) can be improved by presenting it as a logical pipeline, numbering and naming each stage (e.g., Stage 1: Identification of relevant variables, Stage 2: Identification of the minimum set of indicators, etc.). Then, describe these stages in the text using exactly the same names as in Figure 1.

 Results

 8. Elaborate the results according to the stages defined in the Methods section. Follow the same structure and add a framework showing the outputs for each stage and step.

 9. In the paragraph "Literature review of key parameters to be addressed during the first 100 days of an epidemic", the authors use both "35" and "Thirty-five". Please standardize the format: use numerals for numbers 10 and above, and words for numbers zero through nine.

 10. In the paragraph "Global.health data schema core variables", the authors use inconsistent formatting for category names (e.g., Laboratory Information vs. Medical history). Please standardize the formatting.

 11. Include more details about the Mpox data ingestion in the Democratic Republic of Congo. What was learned from that experience? Please add a paragraph discussing the practical feasibility.

 Discussion

 12. The authors mention that the Global.health core schema may not be comprehensive for all pathogen groups. Please provide an example of a pathogen for which the proposed schema might be insufficient.

 13. The manuscript mentions that language could be a barrier for non-native English speakers. How was this challenge addressed in the Democratic Republic of Congo?

Is the work clearly and accurately presented and does it cite the current literature?

Yes

If applicable, is the statistical analysis and its interpretation appropriate?

Not applicable

Are all the source data underlying the results available to ensure full reproducibility?

No source data required

Is the study design appropriate and is the work technically sound?

Yes

Are the conclusions drawn adequately supported by the results?

Yes

Are sufficient details of methods and analysis provided to allow replication by others?

Yes

Reviewer Expertise:

statistics, health data, development and humanitarian analysis

I confirm that I have read this submission and believe that I have an appropriate level of expertise to confirm that it is of an acceptable scientific standard, however I have significant reservations, as outlined above.

# Response

**Published:** 2026-03-21

**Authors:** Everlyn Kamau

**Affiliations:** University of California San Francisco, San Francisco, California, USA

**Reviewer 1** Dear Editorial Team, dear authors,

 Thank you very much for the opportunity to review the paper entitled "Unified framework for the ingestion of early epidemic data for downstream data analytics." I would like to sincerely commend the authors for their effort and dedication. The topic addressed is highly relevant and impactful.

 Overall, the paper presents a proposal for the collection of data during the early phase of a disease outbreak by establishing a unified schema that captures the most relevant indicators for decision-making. I believe the manuscript is suitable for indexing after a medium revision. In particular, I have the following comments:

**We thank the reviewer for their positive assessment of our work.**

 Abstract

 1. The goal of the paper is not mentioned. Please add it in the background subsection. **Thank you. We have added the goal of the paper in the abstract and background section. **  Methods

 2. The description of the methods could be improved. Briefly describe Figure 1, indicating that it represents a methodology based on different steps. **Thank you. The methods section has been reorganized and rewritten for clarity. Figure 1 has been revised and described for clarity.**

 3. The indication that the early phase refers to the first 100 days should be introduced earlier in the text, ideally at the first instance it is mentioned, to ensure clarity for the reader.

**Thank you. This has been clarified by moving the introduction of this term earlier in the introduction section and defining it at its first instance.**

 Introduction

 4. Please add a paragraph about the impact of diseases and the relevance of studying them. For instance, include the number of deaths from COVID-19 using data from the WHO COVID-19 dashboard or the number of disease outbreaks worldwide from this paper: https://doi.org/10.1038/s41597-022-01797-2

**Thank you. While useful information for any health-related research, the authors do not consider the additional value of such information in the context of the current work.**   5. Define the concept of data interoperability. **Thank you. Data interoperability implies the seamless access, exchange, integration, and cooperative of use of data in different systems, applications, and organizations in a coordinated way, ensuring meaning, integrity, and usefulness are maintained. This definition has been added to improve the introductory section**.

 6. Add a paragraph describing the goal of the paper and its structure. **Thank you. The last paragraph in the introduction now describes the goal and structure of the paper.**   Methods

 7. Figure 1 (Conceptual framework of this study) can be improved by presenting it as a logical pipeline, numbering and naming each stage (e.g., Stage 1: Identification of relevant variables, Stage 2: Identification of the minimum set of indicators, etc.). Then, describe these stages in the text using exactly the same names as in Figure 1.

**Thank you. Figure 1 has been updated to improve clarity per your suggestions.**

 Results

 8. Elaborate the results according to the stages defined in the Methods section. Follow the same structure and add a framework showing the outputs for each stage and step. **Thank you. The results section has been restructured to match the sections of the methods. Outputs have been detailed in each Table to show the outputs of the steps.**

 9. In the paragraph "Literature review of key parameters to be addressed during the first 100 days of an epidemic", the authors use both "35" and "Thirty-five". Please standardize the format: use numerals for numbers 10 and above, and words for numbers zero through nine. **Thank you. Use of words and figures for numbers has been standardised.**

 10. In the paragraph "Global.health data schema core variables", the authors use inconsistent formatting for category names (e.g., Laboratory Information vs. Medical history). Please standardize the formatting. **This has now been addressed - thank you.**

 11. Include more details about the Mpox data ingestion in the Democratic Republic of Congo. What was learned from that experience? Please add a paragraph discussing the practical feasibility. **Thank you. That information is beyond the scope of this paper and is under consideration for publication in a separate work. We removed mention of specific outbreaks.**

 Discussion

 12. The authors mention that the Global.health core schema may not be comprehensive for all pathogen groups. Please provide an example of a pathogen for which the proposed schema might be insufficient. **Thank you. We are not aware of an actual example of a pathogen for which the proposed schema might be insufficient. We have provided Disease X as an example and suggested ways in which it may require variables outside of the current schema.**

 13. The manuscript mentions that language could be a barrier for non-native English speakers. How was this challenge addressed in the Democratic Republic of Congo? **Thank you. In the DRC the primary language for ingestion was French and we used translation services and large language models to translate from French to English. While we managed this situation, languages with more complex translation requirements or different characters may not be so manageable.**
